# Protective manifestation of herbonanoceuticals as antifungals: A possible drug candidate for dermatophytic infection

**DOI:** 10.1002/hsr2.775

**Published:** 2022-08-10

**Authors:** Anusha Sharma, Sarika Gupta

**Affiliations:** ^1^ Department of Bioscience and Biotechnology Banasthali Vidyapith Banasthali Rajasthan India

**Keywords:** antifungal activity, fungal dermatophytic, herbalnanoceuticals and green synthesis, medicinal plants

## Abstract

**Background and Aims:**

Fungal dermatophytosis or Tinea is a predominance in about 20%–25% of all total world populations. Dermatophyte infections are mainly caused by fungi belonging to Trichophyton, Epidermophyton, and Microsporum genera along with some other fungi. This epidemiological distribution may change with migration, lifestyle, immunosuppressive state, drug therapy, and socioeconomic conditions.

**Methods:**

The present review indicated the bioefficacy of herbal and herbonanoconjugate as safe management of fungal dermatophytic infection.

**Results:**

It also emphasized the action mechanism as fungicidal and fungistatic with different harmful impacts indicating the need for alternative therapeutics. Simultaneously, the herbal and herbonanoconjugate approaches proved better to manage the prevalence of hepatotoxicity, nephrotoxicity, nausea, altered taste, anemia, GI upsets, hair loss, and so forth. due to conventional oral treatment approaches.

**Conclusion:**

Adoption of the remedial approach can be recommended after preclinical trials' approval as a safe treatment.

## 1 INTRODUCTION

**Figure 1:**
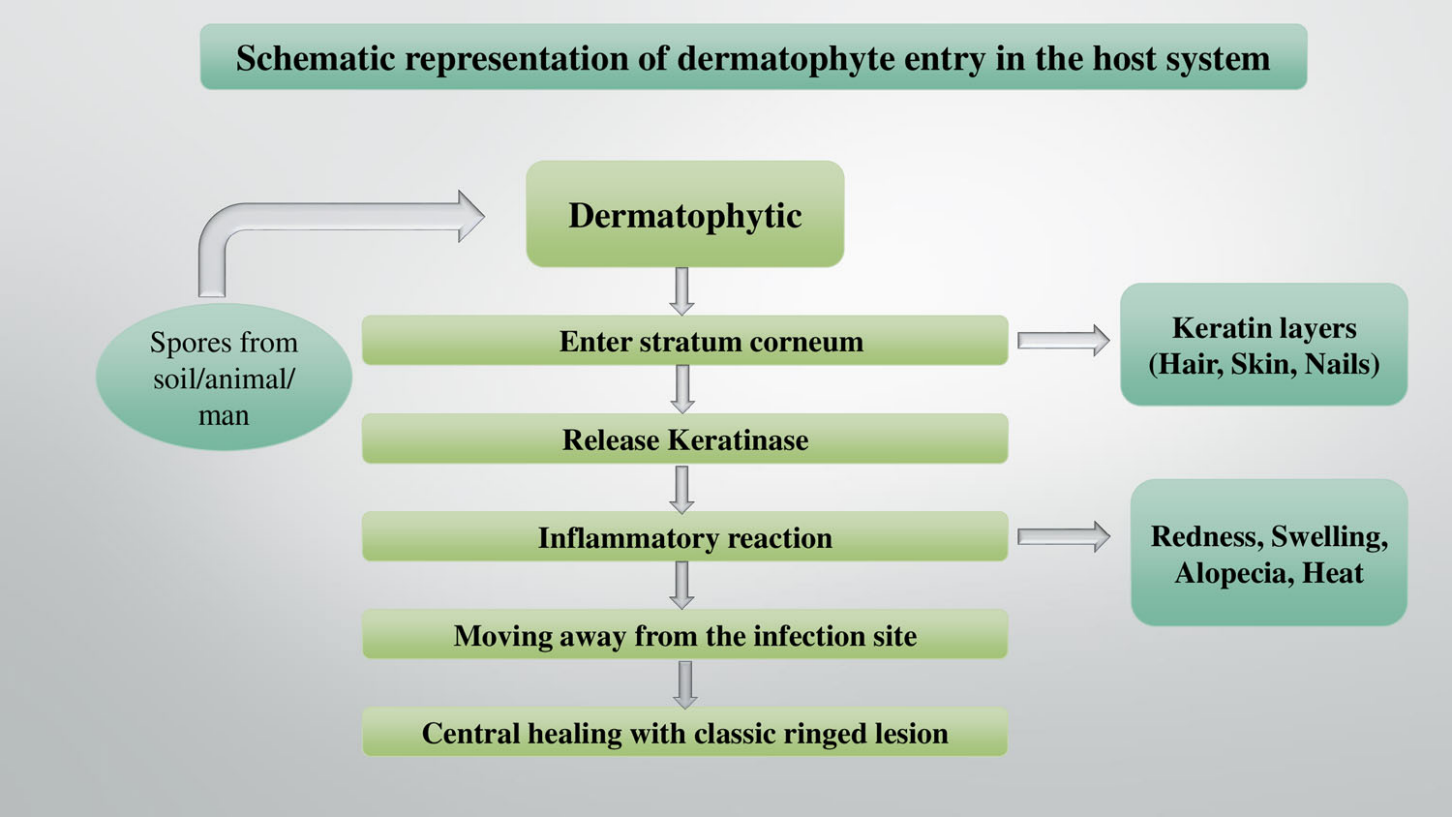
The schematic way in which dermatophytes enter the host system

The term “keratinolytic” is used for fungi inculcated with the capabilities to attack and use keratin with the aid of enzymes. Nails, skin cells, and hair are the parts of the human body that are rich in keratin content. Dermatophytes are groups of closely related filamentous fungi whose infections are confined to superficial keratinized tissues of the hair, skin, and nails. Figure 1.

Due to the fungal infection peg's inability to enter deeper tissues or immune‐competent organs, it remains restricted to epidermal and sub‐epidermal layers of the skin. It cannot reach the deeper tissues or organs of immunocompetent hosts; infection is usually cutaneous and limited to the nonliving cornified layers. Due to the host's sensitivities to the fungal metabolites, the severity of the infecting ranges from moderate to severe affecting the anatomic location in response to the local environmental conditions.^1^, ^2^, ^3^

The etiologic agents of dermatophytosis are divided into nine Deuteromycota (Fungi imperfecti) genera: *Epidermophyton* sp., *Microsporum* sp., and *Trichophyton* sp.^1^

The genera follow the classification system of the Emmons (1934)^31^ scheme based on conidial morphology and its formation, they belong to various genera as *Epidermophyton floccosumm* with soft, thin‐medium thick walls and 1–9 septa, 20–60 in number, 4–13 mm apart, and the macroconidia are widely clavate. They are generally plentiful and borne singly or in clusters. To date two species of this genus are known and among which only one is pathogenic that is *E. floccosum, Microsporum audouinii* is the type of species whose macroconidia is characterized by rough walls which can be verrucose, echinulate, or asperulated (Table 1). *Trichophyton* sp. has soft and generally narrow walls and when macroconidia are present possession of 1–12 septa.^1^

**Table 1:** Salient features of dermatophytosis

Clinical type	Synonym	Site of infection	Causative agents	Symptoms	References
*Tinea barbae*	Barber's itch	Beard, mustache hair	*Microsporum canis*, *Trichophyton tonsurans*, *Trichophyton violaceum*	Like *tinea capitis* often with deep folliculitis	^[^ ^4^ ^]^
*Tinea capitis*	Roundworm	Scalp hair	*M. canis*, *T. tonsurans*, *T. violaceum*	Noninflammatory lesions characterized by patches of alopecia with scaling and broken hairs. Inflammatory lesions show tender boggy swelling	^[^ ^5^ ^]^
*Tinea corporis*	Ringworm	Nonhairy skin of the body	*Trichophyton mentagrophytes*, *Trichophyton rubrum*, *T. violaceum*	Erythematous scaly annular plaques	^[^ ^6^, ^7^ ^]^
*Tinea cruris*	Jock itch, dhobi itch	Nonhairy skin of the groin	*T. mentagrophytes*, *T. rubrum*	Erythematous scaly annular plaques	^[^ ^7^, ^8^ ^]^
*Tinea faciei*	Kerion	Nonhairy skin of the face	*Epidermophyton floccosum*, *T. mentagrophytes*, *T. rubrum*	Erythematous scaly annular plaques	^[^ ^4^ ^]^
*Tinea manuum*	Hands	Palms	*Trichophyton interdigitale*, *T. rubrum*	Diffuse scaling of palm	^[^ ^2^ ^]^
*Tinea pedis*	Athlete's foot	Feet	*T. interdigitale*, *T. rubrum*	Fissuring, scaling, or maceration in the interdigital or sub digital areas	^[^ ^9^ ^]^
*Tinea unguium*	Onychomycosis	Fingers, toenails	*T. interdigitale*, *T. rubrum*	Subungual scaling and lifting of the distal nail	^[^ ^10^ ^]^

*Note*: *Epidermophyton* sp.: *E. floccosum*; *Microsporum* sp.: *M. canis*; *Trichophyton* sp.: *T. mentagrophytes*, *T. rubrum*, *T. interdigitale*, *T. violaceum*.^4^, ^5^, ^6^, ^7^, ^11^, ^12^, ^13^, ^14^

Dermatophytosis is a *Tinea* infection in addition to the ringworm. In humans, the classification of *Tinea* depends on the body part it affects, as “*Tinea capitis*” is an infection usually induced by members of the *Trichophyton* sp. and *Microsporum* sp. It also involves the skin surface and hair as the site of infection. “*Tinea corporis*” generally occurs on the trunk, limbs, and other glabrous skin. “*Tinea cruris*” is responsible for chronic and acute infection of the groin and adjacent parts including the penis and scrotum. It is also an infection of the groin and perianal area and seldom on the upper thighs in adult men, *E. floccusm*, and *T. rubrum* are the most used agents. “*Tinea pedis*” (Athlete's foot) is an infection of the foot (especially soles and toe webs) resulting due to *T. mentagrophytes* infection. Most frequently occurring chronic agents also include *T. rubrum*, *E. floccosum*, and *T. mentagrophytes* var. *interdigitale*. Dermatophyte infection in the nail called onychomycosis includes the toe and fingernails is called *Tinea unguium*. The main familiar dermatophytes of this infection are *T. rubrum* and *T. mentagrophytes*.^1^

### 1.1 Pathophysiology—Symptoms

Primary symptom of dermatophytosis includes itching at the infection site of the human body. In the infection site, *Tinea corporis* reveals severe itching, also in *T. cruris*, itching can be painful if further skin maceration is caused by sweating. It is frequent scratching of *T. cruris* makes it known as jock itching to scratch the skin because of the disease's constant stimulation.^8^ Certain forms of *Tinea* show different rates of odor that can release a very distinctive smell from the infection site. This symptom is the result of the macerated skin cells that are enclosed between the figures in high humidity levels, particularly in the foot (Table 2). In onychomycosis or nail infection, the nail's appearance can change from normal and bright to dull, opaque yellow, thickened, brittle, and crumbling infection.^9^

**Table 2:** Recommended dosing of different systemic antifungal in dermatophytosis

Class	Drug	Preparation	Disease state	Dosage	Target of organism	Medicinal property	References
Azoles (Imidazoles)	Clotrimazole	Cream, gel, lotion, solution, powder	*Tinea versicolor*	4–5 (troches) once daily for 10–14 days	*Aspergillus* sp., *Malassezia furfur*	Anti‐inflammatory, anticancer, antifungal, antiviral, wound healing	^[^ ^15^ ^]^
	Ketoconazole	Cream, lotion, shampoo, soap, powder	*Tinea pedis*, *Tinea cruris*, *Tinea corporis*	2% Cream apply once daily 2 weeks Oral 200–400 mg/once daily for 4 weeks	*C. albicans*, *M. furfur*	Antiviral, antifungal	^[^ ^16^ ^]^
	Miconazole	Cream, lotion	Oral candidiasis, canine *Malassezia dermatitis*	50‐mg once in daily for 10‐14 days	*Aspergillus* sp.	Antibacterial, antiprotozoal	^[^ ^17^ ^]^
Azoles (Trinazoles)	Fluconazole	Cream, Gel, Lotion, Powder, Solution	*Candida* sp.	Oral: 150–300 mg once weekly 2–4 weeks	*Candida* sp.	Antiviral, antifungal	^[^ ^18^ ^]^
	Itraconazole	Cream	*Tinea unguium*, *Pityriasis versicolor*	200 mg/day in two times daily 1–2 weeks	*A. fumigatus*, *A*.*terreus*, *C*. *neoformans*	Antiviral, antifungal	^[^ ^19^ ^]^
	Efinaconazole	Solution	*Tinea pedis*, *Tinea unguium*	One troche dissolved in the mouth five times a day for 2 weeks	*Aspergillus* sp., *T. mentagrophytes*, *T. rubrum*	Antiviral, antifungal	^[^ ^20^ ^]^
Allylamines	Naftifine	Cream, gel, solution, powder	*Tinea pedis* (athlete's foot)	*1% cream in Twice daily for* 15 days	*E. floccosum*, *T. mentagrophytes*, *T. rubrum*, *T. tonsurans*, *M. audouini*, *M. cani*s, *M. gypseum*	Antifungal	^[^ ^21^ ^]^
	Terbinafine	Cream, gel	*Tinea capitis*, *Tinea corporis*, *Tinea* pedis, *Tinea unguium*	250 mg once daily for 1 week every 1 month	*T. rubrum*, *T. tonsurans*	Antifungal, anti‐inflammatory	^[^ ^19^ ^]^
Benzylamines	Butenafine	Cream	*Tinea capitis*, *Tinea corporis*, *Tinea pedis*	1% (Mentax) cream 1 week or once daily for 4 weeks	*T. rubrum*.	Antifungal, antiviral	^[^ ^22^ ^]^
Polyenes	Amphotericin B	Lipid based gel	*Tinea corporis*	1 mg/kg once daily for 2–4 weeks.	*H. capsulatum*, *B. dermatitidis*, *C. neoformans, Candida* sp., *A. fumigatus*, *A. flavus*	Antifungal	^[^ ^23^ ^]^
	Nystatin	Cream	Oral thrush, intestinal infection, vaginal infection, cutaneous infection	4–5 per daily for 10–14 days	*Candidemia*, Invasive *aspergillus*	Antifungal	^[^ ^19^ ^]^

*Note*: *Aspergillus* spp.: *A. flavus*, *A. fumigates*, *A. terreus*; *Blastomyces* sp.: *B. dermatitidies*, *Candida* sp.: *C. albicans*; *Cryptococcus* sp.: *C. neoformans*; *Epidermophyton* sp.: *E. floccosum*; *Histoplasma* sp.: *H. capsulatum*; *Malassezia* sp.: *M. furfur*; *Microsporum* spp.: M. audouini, *M. canis*, *M. gypseum*; *Trichophyton* spp.: *T. interdigitale*, *T. mentagrophytes*, *T. rubrum*, *T. tonsurans*, *T. violaceum*).^15^, ^16^, ^17^, ^18^, ^24^

Manifestations of ringworm by area on the body:

1. Feet (athlete's foot or “competitor's foot”). The indications of ringworm on the feet incorporate red, swollen, stripping, and irritated skin between the toes (particularly between the pinkie toe and the one close to it). The underside and impact point of the foot may likewise be influenced. In serious cases, the skin on the feet can rankle.^25^
2. Scalp (fungus capitis): Ringworm on the scalp typically resembles a layered, bothersome, red, round uncovered spot. The bare spot can develop in size and numerous spots may create if the disease spreads. Ringworm on the scalp is more typical in youngsters than it is in grown‐ups.^26^
3. Groin (fungus cruris or “athlete tingle”): Ringworm on the crotch looks like layered, irritated, red spots, for the most part on the internal sides of the skin folds of the thigh.^25^, ^27^
4. Beard (mouth fungus): Side effects of ringworm on the facial hair incorporate textured, irritated, red spots on the cheeks, jawline, and upper neck. The spots may get crusted over or loaded up with discharge, and the influenced hair may drop out.^28^

### 1.2 Diagnosis

**Figure 2:**
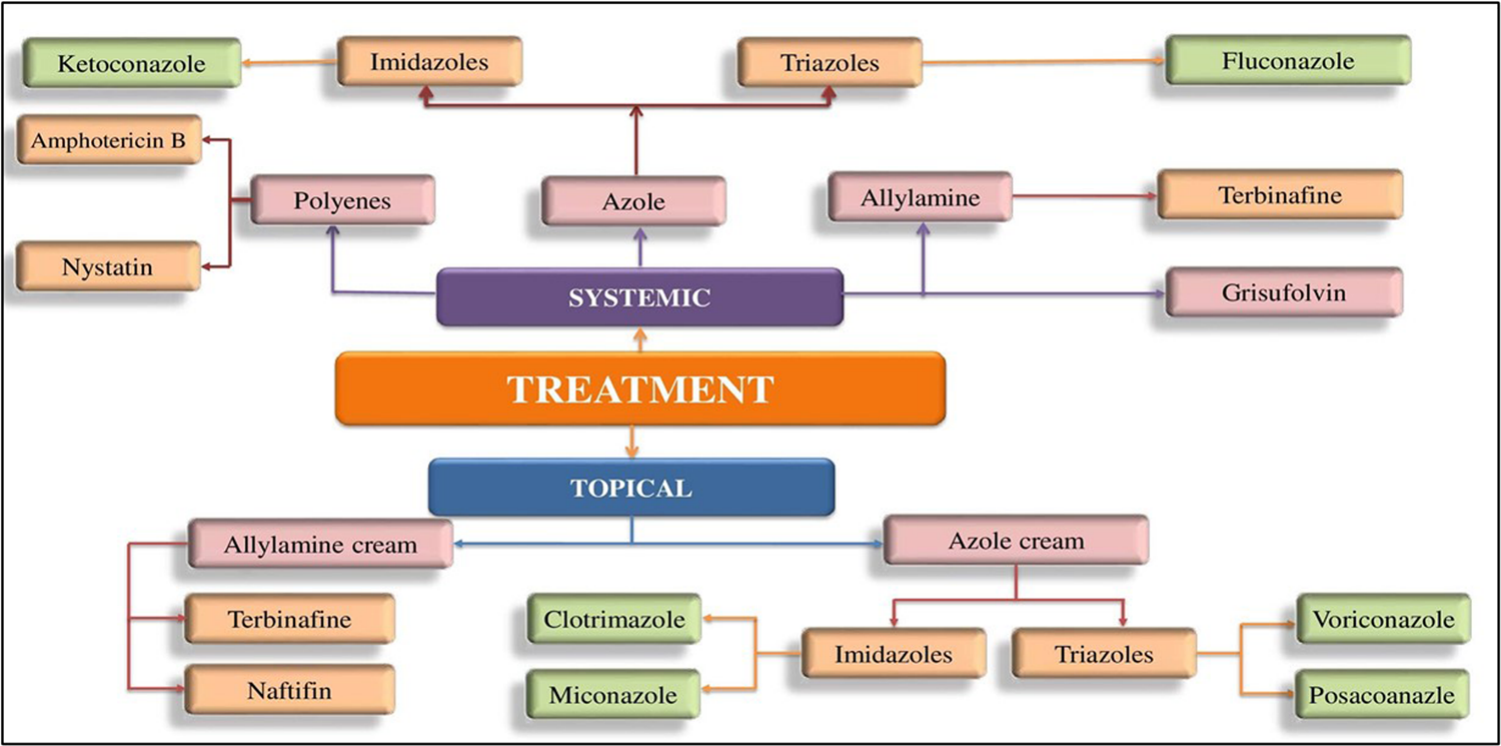
Conventional treatment role of topical antifungals and systemic antifungals

In line with progression in mycological diagnostic approaches, there are two diagnostic categories in today's scenario in traditional and advanced molecular diagnostic aspects. Diagnostic procedures are important features for accuracy, availability, speed, sensitivity, specificity, and cost‐effectiveness (Figure 2). However, traditional routine diagnostic tools cannot guarantee adequate sensitivity and specificity.^29^

#### 1.2.1 Traditional methods of diagnosis

Traditional mycological diagnosis involves various methods as

1. *Specimen direct microscopy*: Direct microscopy is the diagnostic method that is cost‐effective and consumes less time in the observation of fungi in clinical samples. It is mandatory to prepare the clinical specimen by scalpel, poured on a glass slide; further a drop of 10%–20% KOH is added. Based on an earlier study it is concluded that Direct KOH microscopy sensitivity and specificity are ∼65% and >45%.^29^, ^30^
2. *Wood's test lamp*: Wood's lamp tool is a diagnostic method that is helpful to detect *Tinea capitis* caused by *Microsporum canis* (blueish green fluorescence) and *M. audouinii* (greyish yellow fluorescence) dermatophytes in the dark room.^29^
3. *Medium of fungal culture*: Culture is a costly and time‐inefficient strategy with higher specificity and low sensitivity (30%–35%).^29^, ^30^
4. *Biopsy*: Skin or nail biopsy is done when dermatophytosis clinical manifestations occur, however, the findings of culture and microscopy are negative, and therapy does not respond to ringworm.^31^

#### 1.2.2 Advanced molecular diagnosis

It provides successful approaches with high precision, sensitivity, and specificity for the diagnosis of pathogenic dermatophytes. Extraction of DNA molecules from the collected clinical samples and their amplification by universal primary sets targeting 18S rDNA and internal transcribed spacer (ITS) regions in molecular diagnostic methods is performed.^29^ The ITS regions are species‐specific for *Microsporum* sp., *Epidermophyton* sp., and *Trichophyton* sp. Polymerase chain reaction (PCR), conventional real‐time PCR, nested‐PCR, multiplex PCR, DNA microarray, random amplified polymorphic DNA, amplified fragment length polymorphism, restriction fragment length polymorphism, and other nucleic acid‐based techniques give a species‐level diagnostic approach that is quicker, easier and more confident, and can lead to efficient treatment at the onset of dermatophytosis.^29^

### 1.3 Treatment Approaches

#### 1.3.1 Conventional treatment role of topical antifungals

Topical or oral antifungal medicines, or a combination of both, are used to treat dermatophytosis, depending on the severity and extent of the infection, the location of the illness, and the causative organism. Basic, superficial dermatomycoses, are often regarded as first‐line therapy due to their great efficacy and low potential, as well as related undesirable effects.^18^ Depending on the site of the infestation, these medications are blended into various vehicle types, such as lotions, creams, sprays, or gels, to aid penetration and efficacy.^31^ Then applied to the outer area of the skin, they quickly penetrate the stratum corneum, functioning as fungicidal/fungistatic agents for mycological eradication. Polyene, azoles, and allylamine/benzylamines are the most common antifungal medicines used on the skin (Table 3). Several novel antifungal medications with greater effectiveness and anti‐inflammatory properties have lately been launched in India, expanding the arsenal against these chronic dermatoses.^31^

**Table 3:** Classification and mechanism of action of antifungal antibiotics^18^, ^32^, ^33^, ^34^

Class	Drug	Mechanism of action	Mode of action	Side effects	References
Azoles (Imidazoles)	Clotrimazole	Inhibition of fungal lanosterol 14 demethylase causes ergosterol depletion then sterol build‐up in the fungal cell membrane, which is dangerous	Fungistatic, fungicidal	Local irritation, allergic reactions	^[^ ^34^ ^]^
	Ketoconazole			Hepatotoxicity	^[^ ^19^ ^]^
	Miconazole			Liver damage, nausea, arrhythmias, pruritus, hyponatraemia, hyperlipidaemia, dyscrasias	^[^ ^34^ ^]^
Azoles (Triazoles)	Fluconazole	Ergosterol production is inhibited, resulting in a decrease in the integrity and activity of fungal cell membranes	Fungistatic, fungicidal	Hepatic toxicity, increased plasma concentrations of (topical/oral) anaphylaxis, stevens‐Johnson syndrome, hypokalaemia, exfoliative skin disorders, agranulocytosis, thrombocytopenia, toxic epidermal necrolysis, adrenal cortex dysfunction	^[^ ^34^ ^]^
	Itraconazole			Liver function (but hepatotoxicity is milder than ketoconazole, and hypokalaemia with hypertension is caused by corticosteroids with aldosterone‐like action accumulating. Nausea, epigastric discomfort, headache, and edema are all symptoms of leukopenia. Causing heart failure in patients who are elderly or on calcium channel blockers	^[^ ^19^ ^]^
	Efinaconazole				
				Oral burning, xerostomia, altered taste, minor GI‐upset followed by oral ingestion of the drug, allergic reactions	
Allylamines	Naftifine	Squalene epoxidase inhibition: activity linked to squalene accumulation's harmful consequences	Fungistatic	Burning, erythema, dryness, itching	^[^ ^35^ ^]^
	Terbinafine	It inhibits squalene epoxidation, a key initial stage in the formation of ergosterol, by functioning as a noncompetitive inhibitor of fungal squalene epoxidase	Fungistatic, fungicidal	Hepatobiliary dysfunction, induction, agranulocytosis, exacerbation of lupus, severe skin reactions	^[^ ^36^ ^]^
Benzylamines	Butenafine	Interferes with ergosterol production by blocking the enzyme squalene 2, 3 epoxidase, which converts squalene to squalene oxide	Fungicide		^[^ ^16^ ^]^
Polyenes	Amphotericin B	It's an antifungal that's classified as “polyene.” Polyenes bond to ergosterol found in fungi (the primary sterol in fungal cell membranes). The permeability of the cell membrane is altered, and intracellular components seep out of the cell. Depending on the level of concentration in the body	Fungistatic, fungicidal	Serum creatinine, complete blood counts, serum magnesium, blood urea nitrogen, serum sodium, serum potassium, liver function test result must be monitored. When it is used with amino glycosides or neuromuscular blockers, prolonged skeletal muscle paralysis	^[^ ^19^ ^]^
	Nystatin	It's a polyene‐based antifungal that exclusively effects on *Candida*	Fungicidal	GI‐ upset is common when nystatin is taken orally	^[^ ^19^ ^]^

Although the data was limited, nafitine (1%) was found to be superior to placebo in terms of mycological cure rates. Azole combinations with corticosteroids were somewhat more successful than azoles alone in terms of clinical cure, but there was no statistically significant difference in mycological cure. This might be owing to the inflammatory component and symptoms improving quickly, resulting in enhanced patient compliance. Inadvertent use of this combo medication, on the other hand, has been linked to treatment failure and deleterious consequences after steroid misuse, as well as a particular cutaneous variant known as *Tinea pseudoimbricata*.^18^

#### 1.3.2 Role of systemic antifungals

In case of severe intervention or patients who fail topical treatment, systemic antifungal is indicated. Oral treatments have been used for treating onychomycosis, *Tinea capitis* along with the following topical agents as amorolfine/ciclopirox.^22^ There are five main agents of the system: ketoconazole, terbinafine, fluconazole, itraconazole, griseofulvin, and so forth. The most frequently used drug for onychomycosis is the oral dosage of terbinafine and itraconazole. Griseofulvin plays a significant role in the treatment of *Tinea capitis*, although it has been replaced by itraconazole and terbinafine in other regions.^23^

### 1.4 Herbal cure

According to the World Health Organization (WHO), medicinal plants would be the best source for a range of pharmaceuticals, and 80% of the world's population relies on traditional medicine, with a substantial number of traditional therapies requiring the use of plant extracts or active substances.^35^ India has approximately 18,000 angiosperm plants, of which approximately 2500 species are considered a major source of medicinal and aromatic chemicals. Due to their skin‐friendliness and lack of side effects, the market for herbal medicines is growing rapidly.^35^ Medicinal plants have made a substantial contribution to the preservation of human health and hence to the improvement of society's quality of life. In recent times, there has been a greater emphasis on plant study across the world, and a vast body of data has accumulated to demonstrate the enormous potential of medicinal plants employed in diverse traditional systems.^36^ Many herbs are used to treat cardiovascular difficulties, liver illnesses, central nervous system disorders, digestive problems, and metabolic disorders. They can be used as a medicine or supplement in the treatment/management of a variety of disorders due to their ability to provide significant therapeutic effects. Herbal medications or therapeutic plants, as well as their extracts and isolated compound(s), have shown a wide range of biological activity. In folklore, such have been used and continue to be utilized as a medication or dietary supplement for a variety of ailments.^37^ People's eating habits and lifestyles are changing these days; they are consuming junk food, which increases the risk of numerous ailments. Herbs have been utilized for medical purposes since the dawn of mankind. Herbs include active compounds that have anti‐disease properties. In pharmaceutical firms, just the active component is used in the medicine, however, in designer foods, the entire herb extract is included, providing additional benefits.^38^

#### 1.4.1 Plants

Because of their richness and abundance of bioactive phytochemicals and secondary metabolites, plants can be used as possible therapeutic agents against a variety of diseases. Nearly 80% of the world's population uses traditional medicines for basic health care, with plant extracts being utilized in most cases.^39^ Because modern medicine is out of reach for most of the world's population, herbal medicine is becoming a preferred and safe alternative to pharmaceuticals.^40^ Factors such as the convenience with which herbal treatments may be obtained, as well as the availability of practitioners at all times and in all locations, are encouraging. Traditional medicine systems have been based upon the diverse range of flora that processes the natural medicinal properties and have been used for thousands of years for the treatment of various diseases.^41^ Today, according to the reports plant‐based systems still play a vital role in health care. Because of the unrivaled abundance of chemical variety, natural products derived from these medicinal plants, whether as pure compounds or standardized extracts, give limitless prospects for novel therapeutic leads. Interest in edible plants has developed across the world because of the rising demand for chemical variety in screening programs, as well as a desire to find medicinal medications from natural sources.^42^ Plant diseases can be completely controlled using synthetic fungicides, but residual toxicity, pollution, and pathogen resistance need the emergence of new treatment options to address these challenges.^43^ As a result, it is critical to not only enhance existing treatments but also to develop new ones that are beneficial. Medicinal herbs have been shown in several research studies to have antifungal and antibacterial effects (Table 4). Extracts of medicinal plants are not only cost‐effective and have a minimal environmental impact, but they also reduce the health risks associated with synthetic pesticides or fungicides.^44^, ^45^ According to the WHO, 70%–80% of the world's population relies on herb plants.^46^ *C. limon* (Rutaceae), *C. fistula* (Caesalpiniaceae), and *E. camaldulensis* (Myrtaceae) are three different types of medicinal plants that have pesticidal and antifungal properties and may be used to treat a variety of ailments in plants, animals, and people.^47^, ^48^, ^49^

**Table 4:** Patent herbal formulation and their use against skin infection

Patent no.	Title	Used part	Remarks	Used for	References
US 7,714, 183 B2	Honey is used in dressings	Honey buffer	Buffer in conjunction with a variety of medicinal and surgical dressings of the required consistency and viscosity	Honey will be used as a wound healing	^[^ ^50^ ^]^
US 2013/0146481 A1	Bioactive compounds derived from *theacea* plants, as well as manufacture and application methods	Possible plant bioactive components	The innovation is based on isolated bioactive chemicals	To prevent inflammatory behavior, protect skin tissue from UV radiation damage, and normalize skin disorders in mammals	^[^ ^51^ ^]^
US 2013/0323337 A1	A regenerative medication based on a novel herbal combination for wound healing	Extracts obtained from *Curcuma longa*, *Hamiltonia suaveolens*, *Glycyrrhiza glabara*, *Tipha angustifolia*, and *Azadirachta indica*	The present invention is based on a novel, synergistic, and effective herbal composition as a regenerative medicine, which includes a combination of therapeutically effective amounts	To treat would healing	^[^ ^52^ ^]^
WO 2014/147638 Al	A natural wound healing matrix with several functions	Tulsi and Curcumin fragments are extracts with herbal medicinal values to increase their properties and have herbal medicinal values	A multifunctional natural wound‐healing matrix is the subject of this invention. A wound bed consisting of a hydrophilic cotton fabric is the basis for the current concept. One side is covered with zwitterionic chitosan with a low molecular weight, which is implanted on top of the silver nanoparticles created	To treat would healing	^[^ ^53^ ^]^
US 8,709, 509 B2	A regenerative medication composed of herbs for the treatment of wound healing	Extracts obtained from *A. indica*, *G. glabara*, *C. longa*, *H. suaveolens*, *T. angustifolia*, and optionally	This invention discloses a novel, synergistic, and effective herbal composition as a regenerative medicine that includes a combination of therapeutically effective amounts	To treat skin disease	^[^ ^54^ ^]^
EP 2 896 396 A1	Topical wound therapy using an herbal formulation	This breakthrough refers to modern herbal blends that may be used topically antimicrobial agents	To facilitate wound healing, in the form of an emulsifying agent and a product that contains at least one herbal component with analgesic, antifungal, anti‐inflammatory, and antibacterial properties	To cure skin and mucosal lesions	^[^ ^55^ ^]^
WO 2019/078931 Al	Wound‐healing dressing with buckwheat honey and bacitracin	A synthesis or preparation containing a combination of buckwheat honey and bacitracin	The component is gelled in a single encarnation	A method for treating acute and chronic wounds and skin disorders, as well as regenerating skin or dermal tissue in a chronic wound, is the most recent invention	^[^ ^56^ ^]^
US 2019/0201474 A1	Formulation of herbal oils for topical use and medicinal uses	Herbal oil based on *Heterophragma roxburghii* bark extract	Dry gangrene, diabetic gangrene, foot ulcer, wet gangrene, burn wounds, bed sores, snake bite wounds, chronic open wounds, diabetic, and gangrene caused by cellulitis are among the therapeutic conditions for which the disclosed topical herbal oil formulation provides an inexpensive alternative healing therapy	The current disclosure is for a topically applied for treating and curing a range of skin disorders and illnesses, as well as all forms of wounds and other therapeutic circumstances linked with diminished human and animal blood flow	^[^ ^57^ ^]^

#### 1.4.2 Phytochemicals

Phytochemicals are active metabolites that are responsible for the medicinal activity of plants. They supply the plant with organoleptic characteristics and color, as well as nonnutritive compounds that have protected humans from a variety of ailments. They are useful in boosting immune responses and providing protection against a variety of diseases. Alkaloids, flavonoids, saponins, tannin, phenolic compounds, phytosterols, proteins, gums, and lignin are the main constituents. Antifungal, antioxidant, and antibacterial activity are some of the biological characteristics of phytochemicals. These are also linked to a decreased risk of heart disease, ischemic stroke, and other chronic illnesses (eczematous dermatitis, polymorphous light eruption, latex allergy, lupus erythematosus, rosacea, psoriasis, acne, eczema). When compared to routinely used synthetic chemotherapeutic drugs, the most essential attribute of these bioactive elements of plants is that they are more effective with little or no adverse effects.^58^ There are some wild medicinal plants (*Azadirachta indica, Anagallisar vensis, Cuminum cyminum, Capparis spinosa, Inula viscosa, Juglans regia, Plumbago europaea, Phagnalon rupestre, Ruscus aculeatus, Ruta chalepensis, Rosmarinus officinalis, Salvia fruticose*) that have significant antifungal activity against the dermatophytes. These phytochemicals have a wide variety of actions that aid in the enhancement of the immune system and the development of long‐term disease resistance to protect the body from hazardous microorganisms. The therapeutic value of these plants is determined by the chemically vital and active components that have a specific physiological effect on humans.^39^ There is a list of components (alkaloids, flavonoids, phenolics, and terpenoids) that make them effective and establish new vistas for developing novel complexes against infections, leading to the development of new medications. The most significant bioactive components of plants include flavonoids, tannin, phenolic compounds, and alkaloids.^59^, ^60^, ^61^, ^62^

### 1.5 Herbo‐nanoconjugate approach

Nanobiotechnology is an active field of investigation in material sciences where plant and their various products find a crucial use in the synthesis of nanoparticles.^63^ Nanoparticles tend to accumulate in most cases, to prevent aggregation, surface passivator reagents are required. However, when used in large quantities, various synthetic passivators such as mercaptoacetate and thiourea are harmful to the ecosystem. Like chemical and physical approaches, the biological method of synthesizing nanoparticles using microorganisms, enzymes, and crop or seed extract provides numerous advantages in making a valuable contribution to nano‐material science as environmentally friendly technologies.^64^ The efficacy of plant‐based phytochemicals in the overall synthesis and structure of nanoparticles and embedded products of specific nanoparticles is highly attractive, as it provides a significant symbiosis between natural sciences and nanotechnology.^65^ Synthesis of biosynthetic nanoparticles does not need toxic chemicals therefore it has minimal impact on the environment. The synthesis course is mostly taking place at ambient temperature and under medium pressure.^66^ The exploitation of fungi is believed to be potentially interesting as they can resist and bioaccumulate metals and secrete large quantities of enzymes, making it easier to increase the synthesis of nanoparticles.^67^ Biological approach has therefore proven cost‐effective, easier, and more based on a greener approach. The principles of green chemistry have gained considerable prominence in this context; they primarily concern the substitution of chemical products and the enhancement or advancement of processes and technologies to minimize or even remove substances that are toxic to health and the environment.^68^ Silver Nanopartoicle (AgNPs) plant‐mediated synthesis is more advantageous compared to methods that use microorganisms, particularly since they can be improved easily are less biohazardous and do not require the elaborate stage of growing cell cultures.^69^

Antifungal effectiveness of AgNPs against *M. canis*, *Trichophyton mentagrophytes*, and *Microsporum gypseum* will be evaluated. On M. canis, *T*. *mentagrophytes*, and *M. gypseum*, the average minimum inhibitory concentration (MIC) of AgNPs was 200, 180, and 170 ml−1, respectively. Gresofulvin had MICs of 25, 100, and 50 ml−1 in these tests. The AgNPs were shown to be less active than Greseofulvin, although they did have an anti‐dermatophytic impact. *Drosera* sp. and *Dionaea* sp. are two species of *Drosera*. Tissue is a natural source of pharmacologically significant chemicals such glucosides, flavonoids, phenolic compounds, and others that are employed as pharmaceutical substrates. *Drosera* sp. and *Dionaea* sp. extracts in chloroform and methanol were shown to be effective in studies. Tissue has great antimicrobial potential. This study has revealed that secondary metabolites from carnivorous plants are a good basis for AgNPs synthesis and resulting AgNPs possess higher antimicrobial activity than the extracts used for their preparations.^70^

The review proposes an illustrative account indicating the bio efficacy as an alternative approach for the herbal cure and associated silver nanoparticles for the safe management of the fungal dermatophytic infection. These plants act as a reducing agent as well as a capping agent for the cure. This study aims to deliver information on herbonanoconjugate that can be effectively used against fungal dermatophytic infection. It also focuses on the action mechanism of various conventional treatments and their impact on safe herbal remedies. Alternatively, the herbal and herbonanoconjugate approach reduces the incidences of hepatotoxicity, nephrotoxicity, nausea, altered sense of taste, anemia, gastrointestinal upsets, hair loss, and so forth. due to conventional oral treatment methodologies.

#### 1.5.1 Advantages of herbal medicines over the conventional method

Natural sources including plants form the basis of modern medicine and contribute largely to the production of commercial drugs. Every tribal society has a traditional medicine system. Plants have been used as medicines for at least 60,000 years, owing to their potential to create secondary metabolites with a variety of pharmacological effects. Plants are used to make around a quarter of the medications prescribed across the world. More than 1000 firms are active in delivering medical plant products, which has a global industry of $60 billion each year^71^ and an impressive number of modern drugs have been developed from terrestrial plants. The described and accepted number of plant species in the world is about 374,000 including 308,312 vascular plants. Of these, 295,383 are angiosperms.^72^ The largest vascular plant family is Orchidaceae followed by Asteraceae,^73^ but there may be several unexplored and unidentified plant species and thus the large parts of the world are still in need of additional botanical expeditions.^74^ In recent years, the research interest in wild plants is growing due to the emergence of several diseases like AIDS, cancer, Coronavirus, severe acute respiratory syndrome, and so forth. Local and traditional knowledge in isolated places, as well as knowledge among immigrant populations, are now being researched.^75^, ^76^ Around 80% of the world's population relies on herbal medicines, according to,^77^ although demand for herbal medicine is increasing in both established and growing nations.^78^ Traditional medicine is thought to employ 52,885 plants, although the actual number of medicinally effective bioactive metabolites in these plants is unclear.^79^ Medicinal plants found in natural areas are increasingly receiving scientific and economic interest, but we still know very little about the treasure trove that exists among our wild places. Unfortunately, climate change, habitat fragmentation, overexploitation, and the illegal trade in medicinal plants have put around 15,000 of the 52,885 plant species on the verge of extinction. Along with the Royal Botanic Gardens at Kew in the United Kingdom, one out of every five plant species on the planet is endangered.^80^ Experts predict that every 2 years, the Earth loses at least one key therapeutic candidate. Plant‐based remedies dominated human medical practices until around 2 centuries ago. Plant‐based medicine consumption has expanded in the West in recent years, and many developing countries have continued to profit from the strong understanding of medicinal plants. Siddha and Ayurveda medications in India, Kampo Medicine in Japan, Traditional Chinese Medicine, and Unani medicine in the Middle East and South Asia, for example, have been in use for hundreds or even thousands of years and are still utilized by most people.^81^

The herbal products form systems of knowledge and practice that have been transferred over centuries from one generation to another, but such indigenous knowledge is vanishing under the pressures of globalization, including the increasing popularity of Western medicine, cultural changes, and so forth. Attempts are being made to isolate active constituents from natural sources that could be used to treat serious illnesses. Wild plants have abundant properties for the discovery of novel pharmacologically active molecules, mainly because of the environmental stress to which they are subjected.^82^

In this context, many useful drugs from plants were discovered because of scientific follow‐up of well‐known plants used in traditional medicine. Plants will continue to be major sources of drug development, especially as the genomic method gains traction,^83^ and plants' potential to create medicines has been depleted in recent years.^84^ Herbs were the first medications mankind utilized because they create multiple pharmacologically active secondary metabolites. As a result, current scientific approaches should be used to research wild plants to identify their efficacy and potential as a source of novel medications.

## 2 CONCLUSION

The proposed review indicated a comparative evaluation of the antifungal efficacy of partially purified phytochemicals from plant origin and their green nanoparticles. The statistically significant components can further be recommended to produce a formulation possessing antifungal capabilities (after toxicity profiling and preclinical trials) that can be used topically against dermatophytic infection. Current investigation can further serve as an alternative to the chemically synthesized hepatotoxic drugs that are available on the market. The present review aims to gift society with an effective, cost‐effective, reliable, and eco‐friendly approach to the treatment of fungal dermatophytosis.

## AUTHOR CONTRIBUTIONS

This work was carried out in collaboration with both authors. Sarika Gupta provided guidance during the study conduct, designed the study, performed the statistical analysis, and standardized the protocol. Anusha Sharma wrote the first draft of the manuscript, and managed the analyses of the study through managing the literature searches. Both authors read and approved the final manuscript.

## CONFLICT OF INTEREST

The authors declare no conflict of interest.

## TRANSPARENCY STATEMENT

Sarika Gupta or Anusha Sharma affirms that this manuscript is an honest, accurate, and transparent account of the study being reported; that no important aspects of the study have been omitted; and that any discrepancies from the study as planned (and, if relevant, registered) have been explained.

## Data Availability

All authors have read and approved the final version of the manuscript (Sarika Gupta or Anusha Sharma) had full access to all of the data in this study and take complete responsibility for the integrity of the data and the accuracy of the data analysis.
